# Carbapenemase Genes Surveillance in *Pseudomonas aeruginosa* From Domestic Animals: A Five‐Year Study From Brazil’s Midwest Region

**DOI:** 10.1155/vmi/8246667

**Published:** 2026-06-29

**Authors:** Mayara Lima Kawasaki, Jackeliny dos Santos Costa, Ester Santa Vieira, Anna Caroline Barbosa Alves Silva, Camila Regina Alves Vani, Ana Carolina Lemos Caldeira, Ana Rebecca Maia Araujo, Luciano Nakazato, Valeria Dutra

**Affiliations:** ^1^ Laboratory of Microbiology, Veterinary Hospital, Federal University of Mato Grosso (UFMT), Cuiabá, Mato Grosso, Brazil, ufms.br; ^2^ Department of Veterinary Medicine, Federal University of Mato Grosso (UFMT), Av. Fernando Corrêa da Costa, 2367, Boa Esperança, Cuiabá, Mato Grosso, 78060-900, Brazil, ufms.br

**Keywords:** carbapenemase, metallo-beta-lactamases, One Health, resistance gene monitoring, veterinary microbiology, zoonotic transmission

## Abstract

**Background:**

*Pseudomonas aeruginosa* represents a critical antimicrobial resistance (AMR) threat in veterinary medicine, with carbapenemase‐producing isolates posing significant challenges to both animal and human health. The emergence of metallo‐beta‐lactamases (MBLs) and serine carbapenemases in veterinary settings necessitates comprehensive surveillance to monitor resistance gene dissemination.

**Objective:**

This study aimed to conduct genotypic surveillance of carbapenemase‐encoding genes (bla_KPC-2_, bla_SPM-1_, bla_NDM-1_, bla_VIM-2_, and bla_IMP-1_) in *P. aeruginosa* isolates from domestic animals in Brazil’s Midwest region, providing critical epidemiological data for antimicrobial stewardship programs.

**Methods:**

A total of 83 *P. aeruginosa* isolates were collected from various domestic animal species (57 canines, 12 bovines, 7 felines, 2 equines, 2 ovines, 2 avians, and 1 rabbit) between 2017 and 2021. Molecular identification was confirmed via 16S rRNA gene sequencing, and carbapenemase genes were detected using conventional multiplex PCR. Data were analyzed using descriptive statistics.

**Results:**

Carbapenemase genes were detected in 40.96% (34/83) of isolates. The most prevalent gene was bla_KPC-2_ at 34.9% (29/83), followed by bla_SPM-1_ (9.6%, 8/83), bla_NDM-1_ (8.4%, 7/83), bla_VIM-2_ (3.6%, 3/83), and bla_IMP-1_ (1.2%, 1/83). Ear infections were the predominant source (53%, 44/83), particularly in canines. Co‐occurrence of multiple resistance genes was observed in 7.2% of isolates.

**Conclusion:**

This study reveals an alarming prevalence of carbapenemase‐producing *P. aeruginosa* in domestic animals, with bla_KPC-2_ frequencies comparable to those in wild animal populations. The predominance of ear‐derived isolates suggests a potential for community‐acquired transmission. These findings underscore the critical need for robust antimicrobial stewardship and integrated One Health surveillance systems to monitor resistance gene flow between animal and human populations.

## 1. Introduction

*Pseudomonas aeruginosa* is an opportunistic, gram‐negative bacterium that exhibits broad antimicrobial resistance (AMR), causing infections with high rates of morbidity and mortality in both animals and humans [1]. The World Health Organization (WHO) classifies carbapenem‐resistant *P. aeruginosa* as a Priority 1: CRITICAL pathogen. AMR is a global health crisis, and the widespread use of the same classes of antimicrobials in human and veterinary medicine facilitates cross‐resistance and the dissemination of resistant microorganisms [2].

This resistance is often mediated by acquired carbapenemase genes, such as *Klebsiella pneumoniae* carbapenemase (KPC), New Delhi metallo‐beta‐lactamase (NDM), Verona integron‐encoded MBL (VIM), imipenemase (IMP), and São Paulo MBL (SPM) [3, 4]. The genetic plasticity of *P. aeruginosa* allows it to acquire and transfer these resistance genes, making it a formidable challenge in clinical settings [5].

In Brazil, the bla_KPC_ gene is a major concern in human healthcare [6]. While initially associated with *Enterobacterales*, its spread to *P. aeruginosa* is a significant public health threat [7]. Similarly, the endemic nature of bla_SPM-1_ in South America and the global rise of bla_NDM-1_ further complicate the AMR landscape [8, 9].

Given the close contact between humans and domestic animals, there is a pressing need to understand the prevalence of these resistance genes in veterinary isolates. This study provides the first systematic genotypic surveillance of carbapenemase genes in *P. aeruginosa* from domestic animals in the Midwest region of Brazil.

## 2. Materials and Methods

### 2.1. Isolates

A total of 83 *P. aeruginosa* isolates were obtained from clinical samples submitted to the Microbiology Laboratory of a public Veterinary Hospital in Mato Grosso, Brazil, between 2017 and 2021. Samples were first streaked onto both 8% sheep blood agar and MacConkey agar plates. Following this, the plates were incubated at 37°C for a period of up to 72 h. Preliminary identification was based on morphological and biochemical characteristics [10].

### 2.2. DNA Extraction and Molecular Identification

Genomic DNA was extracted using the phenol–chloroform method [11]. All isolates were PCR amplified with primers 27F 5AGAGTTTGATCCTGGCTCAG3′and 1492R 5′TACCTTGTTACGACTT′ and confirmed as *P. aeruginosa* by sequencing the 16S rRNA gene. The sequences were compared against the GenBank database using BLAST.

### 2.3. PCR for Resistance Genes

Conventional PCR was performed to detect the presence of carbapenemase genes (bla_KPC-2_, bla_SPM-1_, bla_NDM-1_, bla_VIM-2_, and bla_IMP-1_). Each 25 μL reaction contained 10 ng of genomic DNA, 4.0 pmol of each primer (Table 1), 0.2 mM dNTPs, 3 mM MgCl_2_, 1x PCR buffer (20 mM Tris‐HCl pH 8.4 and 50 mM KCl), and 1U Taq DNA polymerase. Amplified products were visualized by gel electrophoresis. Representative gel images are provided in the Supporting Information (Supporting Figure 1).

**TABLE 1:** Primers used for the detection of carbapenemase genes.

Gene	Sequence (5’–3′)	Product (bp)	Reference
bla_KPC-2_	F: TGTCACTGTATCGCCGTC R: CTCAGTGCTCTACAGAAAACC	1020	Yigit et al. [12]

bla_SPM-1_	F: CCTACAATCTAACGGCGACC R: TCGCCGTGTCCAGGTATAAC	650	Franco et al. [13]

bla_NDM-1_	F: GGTTTGGCGATCTGGTTTTC R: CGGAATGGCTCATCACGATC	621	Hatrongjit et al. [14]

bla_VIM-2_	F: AAAGTTATGCCGCACTCACC R: TGCAACTTCATGTTATGCCG	865	Yan et al. [15]

bla_IMP-1_	F: TGAGCAAGTTATCTGTATTC R: TTAGTTGCTTGGTTTTGATG	740	Yan et al. [15]

### 2.4. Statistical Analysis

Data were analyzed descriptively, and results are presented as counts and percentage frequencies.

## 3. Results

**FIGURE 1:**
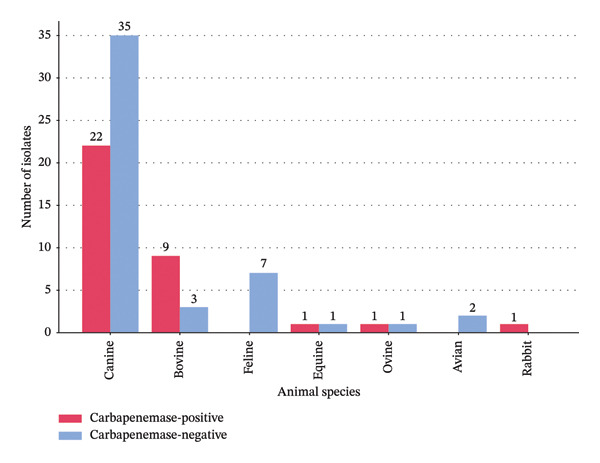
Carbapenemase production in *P. aeruginosa* isolates from various animal species. Stacked bar chart showing the number of carbapenemase‐positive (red) and carbapenemase‐negative (blue) isolates for each animal species.

Of the 83 *P. aeruginosa* isolates, 40.96% (34/83) were positive for at least one carbapenemase gene. The distribution of positive and negative isolates across different animal species is shown in Figure 1. Canines were the most sampled species (*n* = 57), with 22 (38.6%) isolates testing positive. However, proportionally higher rates were observed in bovines (75%, 9/12), and although only one rabbit isolated was obtained, it tested positive for a carbapenemase gene.

**FIGURE 2:**
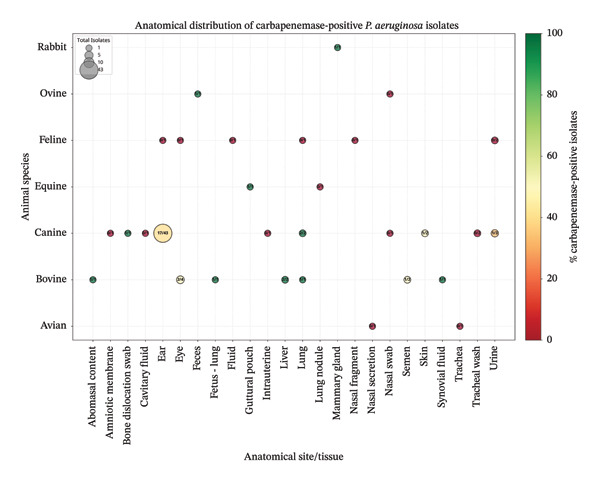
Anatomical distribution of carbapenemase‐positive *P. aeruginosa* isolates. Bubble chart illustrating the distribution of isolates by animal species and anatomical site. Bubble size corresponds to the total number of isolates. Bubble color indicates the percentage of carbapenemase‐positive isolates (red = 0%, green = 100%). The label inside each bubble shows the ratio of positive isolates to the total isolates for that combination.

The anatomical distribution of isolates is detailed in Figure 2. Ear infections were the most common source, with 44 total isolates, of which 17 (38.6%) were positive. Notably, isolates from the liver (2/2) and lung (3/4) showed high positivity rates of 100% and 75%, respectively.

The most prevalent gene was bla_KPC-2_ (34.9%), followed by bla_SPM-1_ (9.6%), bla_NDM-1_ (8.4%), bla_VIM-2_ (3.6%), and bla_IMP-1_ (1.2%). Co‐occurrence of multiple genes was found in 7.2% (6/83) of isolates, with the combination of bla_KPC-2_ and bla_SPM-1_ being the most frequent (4.8%, 4/83), primarily in bovine isolates.

## 4. Discussion

This study provides the first comprehensive surveillance of carbapenemase genes in *P. aeruginosa* from domestic animals in the Midwest region of Brazil, revealing a high prevalence of 40.96%. The predominance of ear‐derived isolates highlights otic infections as a potential reservoir at the human–animal interface and highlights a critical One Health interface due to the close contact between pets and owners [16, 17].

The prevalence of bla_KPC-2_ (34.9%) in our study is alarmingly similar to the 40.7% rate reported in wild animals from the same region, suggesting widespread environmental contamination and circulation of this gene across diverse ecological niches [18]. The detection of the endemically South American gene bla_SPM-1_ (9.6%) and the globally emerging bla_NDM-1_ (8.4%) underscores the complex and interconnected epidemiology of AMR in the region [19, 20].

The co‐occurrence of multiple carbapenemase genes in single isolates indicates complex resistance mechanisms and may suggest the involvement of mobile genetic elements like plasmids, which can accelerate the spread of multidrug resistance. While this study did not include plasmid analysis, these findings highlight the need for future research. Further molecular characterization of these isolates using whole‐genome sequencing would be invaluable to elucidate the genetic context of these resistance genes and understand their transmission dynamics.

## 5. Conclusions

Our findings reveal a high and concerning prevalence of carbapenemase‐producing *P. aeruginosa* in domestic animals in Brazil’s Midwest. The data underscore the urgent need for robust antimicrobial stewardship programs in veterinary medicine and the establishment of integrated One Health surveillance systems to monitor the flow of resistance genes between animal, human, and environmental reservoirs. Future work should focus on the molecular epidemiology of these resistance determinants to inform targeted intervention strategies.

## Funding

The scholarships were funded by Coordenação de Aperfeiçoamento de Pessoal de Nível Superior (CAPES) and the National Council for Scientific and Technological Development (CNPq‐313101/2023‐0).

## Conflicts of Interest

The authors declare no conflicts of interest.

## Supporting Information

Gel electrophoresis analysis of resistance genes.

## Supporting information


**Supporting Information** Additional supporting information can be found online in the Supporting Information section.

## Data Availability

The data that support the findings of this study are available from the corresponding author upon reasonable request.
