# Supplementary material for: Carbapenemase Genes Surveillance in Pseudomonas aeruginosa From Domestic Animals: A Five‐Year Study From Brazil’s Midwest Region
Source: Vet Med Int. 2026 Jun 29;2026:8246667. doi: 10.1155/vmi/8246667 (PMC13315122; doi:10.1155/vmi/8246667)
Supplement: Supplementary file 1 — Supporting Information Additional supporting information can be found online in the Supporting Information section. [file VMI-2026-8246667-s001.pptx]

## Slide 1
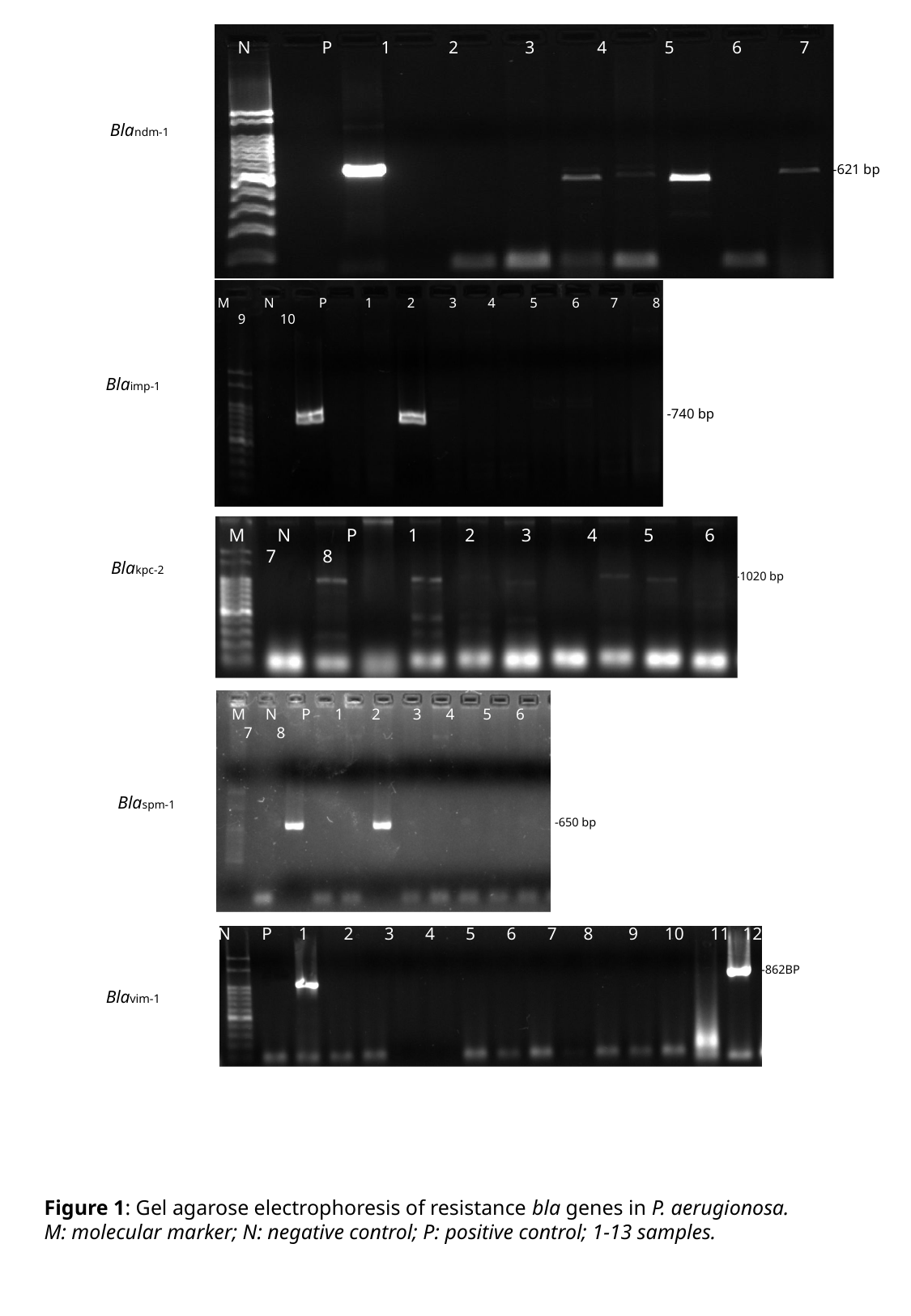

M N P 1 2 3 4 5 6 7 8
-621 bp
Blandm-1
M N P 1 2 3 4 5 6 7 8 9 10
M N P 1 2 3 4 5 6 7 8 9 10
Blaimp-1
-740 bp
M N P 1 2 3 4 5 6 7 8
Blakpc-2
-1020 bp
 M N P 1 2 3 4 5 6 7 8
M N P 1 2 3 4 5 6 7 8
Blaspm-1
M N P 1 2 3 4 5 6 7 8
-650 bp
M N P 1 2 3 4 5 6 7 8 9 10 11 12 13
-862BP
Blavim-1
Figure 1: Gel agarose electrophoresis of resistance bla genes in P. aerugionosa.
M: molecular marker; N: negative control; P: positive control; 1-13 samples.
